# Oral squamous cell carcinoma: microRNA expression profiling and integrative analyses for elucidation of tumourigenesis mechanism

**DOI:** 10.1186/s12943-016-0512-8

**Published:** 2016-04-07

**Authors:** Mayakannan Manikandan, Arungiri Kuha Deva Magendhra Rao, Ganesan Arunkumar, Meenakshisundaram Manickavasagam, Kottayasamy Seenivasagam Rajkumar, Ramamurthy Rajaraman, Arasambattu Kannan Munirajan

**Affiliations:** Department of Genetics, Dr. ALM PG Institute of Basic Medical Sciences, University of Madras, Taramani campus, Chennai, 600113 Tamil Nadu India; Department of Medical Oncology, Government Arignar Anna Memorial Cancer Research Institute and Hospital, Karapettai, Kanchipuram, 631502 Tamil Nadu India; Centre for Oncology, Government Royapettah Hospital & Kilpauk Medical College, Chennai, 600014 Tamil Nadu India

**Keywords:** Oral cancer, Head and neck cancer, Squamous cell carcinoma, microRNA, Microarray, Quantitative PCR, Signaling pathway, Oncogene, Tumour suppressor gene, Interaction network

## Abstract

**Background:**

The advantages and utility of microRNAs (miRNAs) as diagnostic and prognostic cancer markers is at the vanguard in recent years. In this study, we attempted to identify and validate the differential expression of miRNAs in oral squamous cell carcinoma (OSCC), to correlate their expression with the clinico-pathological profile of tumours and to identify the signaling pathways through which the aberrantly expressed miRNAs effect tumourigenesis.

**Methods:**

miRCURY LNA™ array with probes specific to 1168 miRNAs and TaqMan assays specific for 10 miRNAs was employed to evaluate and validate miRNA expression in a discovery cohort (*n* = 29) and validation cohort (*n* = 61) of primary OSCC tissue specimens, respectively. A computational pipeline with sequential integration of data from miRTarBase, CytoScape, UniProtKB and DIANA-miRPath was utilized to map the target genes of deregulated miRNAs and associated molecular pathways.

**Results:**

Microarray profiling identified 46 miRNAs that were differentially expressed in OSCC. Unsupervised clustering demonstrated a high degree of molecular heterogeneity across the tumour samples as the clusters did not represent any of their clinico-pathological characteristics. The differential expression of 10 miRNAs were validated by RT-qPCR (let-7a, let-7d, let-7f and miR-16 were downregulated while miR-29b, miR-142-3p, miR-144, miR-203, and miR-223 were upregulated in OSCC; the expression of miR-1275 was variable in tumours, with high levels associated to regional lymph node invasion; additionally, miR-223 exhibited an association with advanced tumour stage/size). In silico analyses of the experimentally confirmed target genes of miRNAs revamp the relationship of upregulated miRNAs with tumour suppressor genes and of downregulated miRNAs with oncogenes. Further, the differentially expressed miRNAs may play a role by simultaneously activating genes of PI3K/Akt signaling on one hand and by repressing genes of p53 signaling pathway on the other.

**Conclusions:**

The identified differentially expressed miRNAs and signaling pathways deregulated in OSCC have implications for the development of novel therapeutic strategies. To the best of our knowledge, this is the first report to show the association of miR-1275 with nodal invasion and the upregulation of miR-144 in OSCC.

**Electronic supplementary material:**

The online version of this article (doi:10.1186/s12943-016-0512-8) contains supplementary material, which is available to authorized users.

## Background

Oral cancer broadly encompasses tumours arising in the lips, hard palate, upper and lower alveolar ridges, anterior two-thirds of the tongue, sublingual region, buccal mucosa, retromolar trigone and floor of the mouth [1]. Squamous cell carcinoma is the predominant (~95 %) histological type [2] and hence the term ‘oral cancer’ tends to be used interchangeably with oral squamous cell carcinoma (OSCC). In 2012, OSCC accounted for 145,000 deaths worldwide, with less developed regions sharing 77 % of the burden; In India, OSCC is the leading cancer in men and fifth common cancer in women [3]. The widespread practise of smoking or chewing tobacco and alcohol drinking, apart from poor oral hygiene, poor diet and Human Papilloma Virus (HPV) infections may explain this disproportionately higher incidence of OSCC in India [4, 5]. Although the oral cavity is readily accessible for clinical examination, most tumours are not diagnosed until they have advanced or metastasized [6], thereby limiting the effectiveness of chemotherapy, radiotherapy and surgery. Moreover, the development of second primary tumours hamper the success of multimodal therapeutic procedures leading to poor prognosis and dismal 5-year survival rates [3, 7]. Hence, research directed towards the identification of biomarkers for early diagnosis of OSCC, indicators of good or bad prognosis, and determinants of treatment response/overall survival is undeniably essential [8].

MicroRNAs (miRNAs) are short (19-to-25 nt) single stranded non-coding RNAs, that bind to complementary sequences present usually in the 3’ untranslated region (UTR) of target messenger RNAs and inhibit their translation by the subsequent recruitment of RNA induced silencing complex – RISC [9, 10]. Since > 30 % of the human genes are predicted to be regulated by miRNAs, these tiny RNAs govern all cellular, physiological and developmental processes [11]. MicroRNAs are encoded throughout the genome with a vast majority located in intergenic regions (anywhere between 57 and 69 %), followed by intronic regions (~12 to 17 %), exonic (~5 %), long-noncoding (5 %) and repeat regions (~8 %) [12]. Nevertheless, around 50 % of these genomic regions are frequently prone to alterations in various cancers and are collectively termed as cancer-associated genomic regions (CAGRs) [13, 14]. As a consequence, miRNA deregulation is common in all human cancers including OSCC and miR signatures have been helpful at all levels right from diagnosis to determination of treatment response [15]. Majority of the miRNA expression profiling studies performed in OSCC until now represent either oral cancer cell line models [16, 17] or tissue samples of head and neck carcinoma on the whole [18–23]. Although oral, pharyngeal and laryngeal tumours are grouped together as head and neck squamous cell carcinoma (HNSCC), the process of carcinogenesis is quite different leading to molecular heterogeneity [24]. Moreover, variations in risk factors and associated clinical parameters across different geographical areas of the world adds more complexity to OSCC [25]. Hence, studying OSCC separately with sufficient number of primary tumours is crucial to arrive at unifying conclusions. In the present study, we profiled the expression of 1,168 mature miRNAs in 29 OSCC primary tumours by miRCURY LNA™ array and validated the expression of 10 candidates by TaqMan single miRNA assays in a cohort of 61 OSCC samples compared to 9 independent normal oral tissues. We also tested the association of mature miRNA levels with tumour characteristics and elucidated the underlying signaling pathways by an extensive *in silico* analyses pipeline.

## Methods

### Clinical tissue specimens

The study was approved by the Institutional Review Board of Government Arignar Anna Memorial Cancer Research Institute and Hospital – GAAMCRIH, Kanchipuram, (Ref. No.262/E1/2008) and Government Royapettah Hospital – GRH, Chennai, (Ref. No. 371/RMO/2010). Informed consent was obtained from all subjects and samples were collected according to the ethical framework and guidelines of Dr. ALM PG Institute of Basic Medical Sciences, University of Madras, Chennai. The OSCC primary tissues were collected by punch biopsy from patients of GAAMCRIH. The clinico-pathological characteristics like age, sex, the tumour TNM stage, tobacco chewing/smoking status, alcohol consumption, etc. were documented in a standard questionnaire. As a control, normal tissues were obtained from the contra-lateral tumour-free side of OSCC patients attending GRH. All specimens were transferred to sample collection tubes containing 3 mL of RNA*later* solution (Ambion, USA), and transported to the laboratory on ice.

### RNA Extraction and quality control

The equipment used for tissue processing and RNA extraction were subjected to overnight DEPC treatment (0.05 %) and autoclaved. The tissues were removed from RNA*later* upon reaching the laboratory, cut into small pieces, transferred back to the respective tube to facilitate the percolation of RNA*later* and stored at 4 °C for a day. Subsequently, RNA*later* was removed carefully using pipette without any carryover and the tissues were stored at −80 °C. At the time of homogenization, the samples were thawed on ice, transferred to nuclease-free 2 mL microfuge tubes and weighed on an electronic balance (Sartorius, Germany). QIAzol® (Qiagen, USA) was added to the tubes (700 μL per 50–100 mg of tissue) followed by the addition of Zirconia beads of 1 mm diameter (SV Scientific, Bangalore). Homogenization was carried out on Micro Smash MS-100 automated homogenizer (TOMY, Japan) at 3000 rpm for 30 s. The tubes were then allowed to cool on ice for a minute and again homogenized at 3000 rpm for 30 s. This process was repeated three to four times until no tissue blocks were obvious. Finally, the homogenate was incubated for 5 min at room temperature and total RNA was extracted using miRNeasy Mini Kit (Qiagen, USA) as per the manufacturer’s protocol and recommendations. RNA quality was assessed with Agilent 2100 Bioanalyzer (Agilent Technologies, Inc., USA) and was judged by the RNA integrity number (RIN). Only samples with RIN ≥ 5 were included in the array while those with RIN < 5 were excluded (Representative bioanalyzer eletropherogram shown in Additional file 1). RNA concentrations were measured on a NanoDrop ND-1000 (Thermo Scientific, Germany) spectrophotometer.

### MicroRNA microarray

The microarray experiments were conducted at Exiqon Services, Denmark. Total RNA (800 ng) from the sample and the reference was labeled with Hy3™ and Hy5™ fluorescent label, respectively, using the miRCURY LNA™ microRNA Power Labeling Kit, Hy3™/Hy5™ (Exiqon, Denmark). The Hy3™-labeled samples and Hy5™- labeled reference RNA were mixed pair-wise and hybridized to the miRCURY LNA™ microRNA Array (5th gen - hsa, mmu & rno) (Exiqon, Denmark) that contained capture probes targeting all miRNAs for human, mouse or rat registered in the miRBase 16.0. The hybridization was performed according to the miRCURY LNA™ microRNA Array instruction manual using a Tecan HS 4800™ hybridization station (Tecan, Austria). After hybridization, the microarray slides were stored in an ozone free environment (ozone level below 2.0 ppb) to prevent potential bleaching of the fluorescent dyes, scanned using the Agilent G2565BA Microarray Scanner System (Agilent Technologies, Inc., USA) and analysed using the ImaGene 9.0 software (BioDiscovery, Inc., USA). The quantified signals were background corrected [26] (Normexp with offset value 10) and normalized using the global Lowess (LOcally WEighted Scatterplot Smoothing) regression algorithm.

### Reverse transcriptase quantitative PCR

Reverse transcriptase quantitative PCR (RT-qPCR) was performed using TaqMan MicroRNA Reverse transcription kit, TaqMan miRNA assays (Additional file 2) and TaqMan Universal Master Mix II (without UNG). Briefly, cDNA conversion was carried out in a total volume of 15 μL containing 100 mM dNTP, Multiscribe Reverse Transcriptase (50 U/μL), RT buffer, RNase inhibitor, RNA sample (10 ng/15 μL) and 5X TaqMan miRNA specific RT primers. The cDNA was diluted 15-fold, added to 384-well custom plates, and the subsequent PCR used miRNA specific TaqMan assays. A negative control without template was also included in parallel. PCR was carried out on 7900HT Real Time PCR System (Applied Biosystems, Foster City, CA, USA) in a total volume of 10 μL with the following thermal cycling parameters: 95 °C for 10 min once, followed by 40 cycles of denaturation at 95 °C for 25 s and annealing/extension at 60 °C for 60 s. All reactions were carried out in triplicate, the acquired data was analysed using the RQ Manager Software and the resulting text file was exported to Microsoft Excel. The expression of the miRNAs were normalized to the average Ct value of RNU44 and RNU48 (ΔCt). The relative expression of miRNAs in OSCC were calculated using the median ΔCt value of the independent normal oral tissues (calibrator reference) by the 2^-∆∆Ct^ method [27].

### Statistical analysis

For microarray data, unsupervised analysis and supervised clustering analysis of the samples were carried out by the service provider (Exiqon, Denmark). Principle component analysis (PCA) was applied to visualize high-dimensional data generated based on the sample groupings. A student’s *t*-test was performed to identify the miRNAs that significantly differed between the study groups (moderately differentiated and well differentiated histological type of tumours). Association of the clinico-pathological characteristics with the sample clusters were analysed using contingency tables, followed by Fisher’s exact test or Chi-square test as and when appropriate. In case of RT-qPCR, data were analysed using GraphPad Prism 6 (GraphPad software Inc., La Jolla, CA, USA). The fold change ratios of each miRNA was log2 transformed and tested for normality with the D'Agostino & Pearson omnibus test. In case of Gaussian distribution, the difference between the two groups were analysed using Student’s *t*-test. Welch correction was applied post *t*-test, when a significant difference in variance was observed. In case of non-Gaussian distribution, Mann Whitney test for independent samples was applied. All tests were two tailed, and a *P* < 0.05 was considered significant. The relative expression levels are provided as mean with 95 % confidence interval or as median with interquartile range wherever applicable. Association of miRNA levels to the 10 clinico-pathological variables was tested by either Student’s *t*-test or Mann Whitney test as applicable and to account for multiple hypotheses testing, the significance level was adjusted by Bonferroni correction to *P* = 0.005 (0.05/10).

### In silico analyses

From the RT-qPCR data, we constructed a custom miRNA:tumour expression matrix (custom OncoPrint) using the tool “OncoPrinter” available at cBioPortal (URL: http://www.cbioportal.org/oncoprinter.jsp) [28]. The basis and details on how we constructed the custom OncoPrint is described elsewhere [29]. The target genes of miRNAs were retrieved from miRTarBase [30] available at http://mirtarbase.mbc.nctu.edu.tw/, which provides the most current and comprehensive information of experimentally validated miRNA target interactions. The miRNA:mRNA interactions were visualized and analysed using CytoScape® 2.8.3 available at http://www.cytoscape.org [31]. The targets of the downregulated miRNAs were then compared against the list of manually curated and reviewed human ‘oncogenes’ (Keyword: “Proto-oncogene [KW-0656]”; URL: http://www.uniprot.org/uniprot/?query=KW-0656&sort=score), retrieved from the UniProt Knowledgebase, a central hub for the collection of functional information on proteins with accurate, consistent and rich annotation [32]. In a similar manner, the validated targets of over expressed miRNAs were compared against the list of human ‘tumour suppressor genes’ (Keyword: “Tumor suppressor [KW-0043]”; URL: http://www.uniprot.org/uniprot/?query=KW-0043&sort=score) obtained from UniProtKB. DIANA-miRPath v2.0 [33] was used to map the unifying functional pathways of differentially expressed miRNAs (URL: http://diana.imis.athena-innovation.gr/DianaTools/index.php?r=mirpath/index).

## Results

### miRNA profiling asserts the molecular heterogeneity of OSCC

**Fig. 1:**
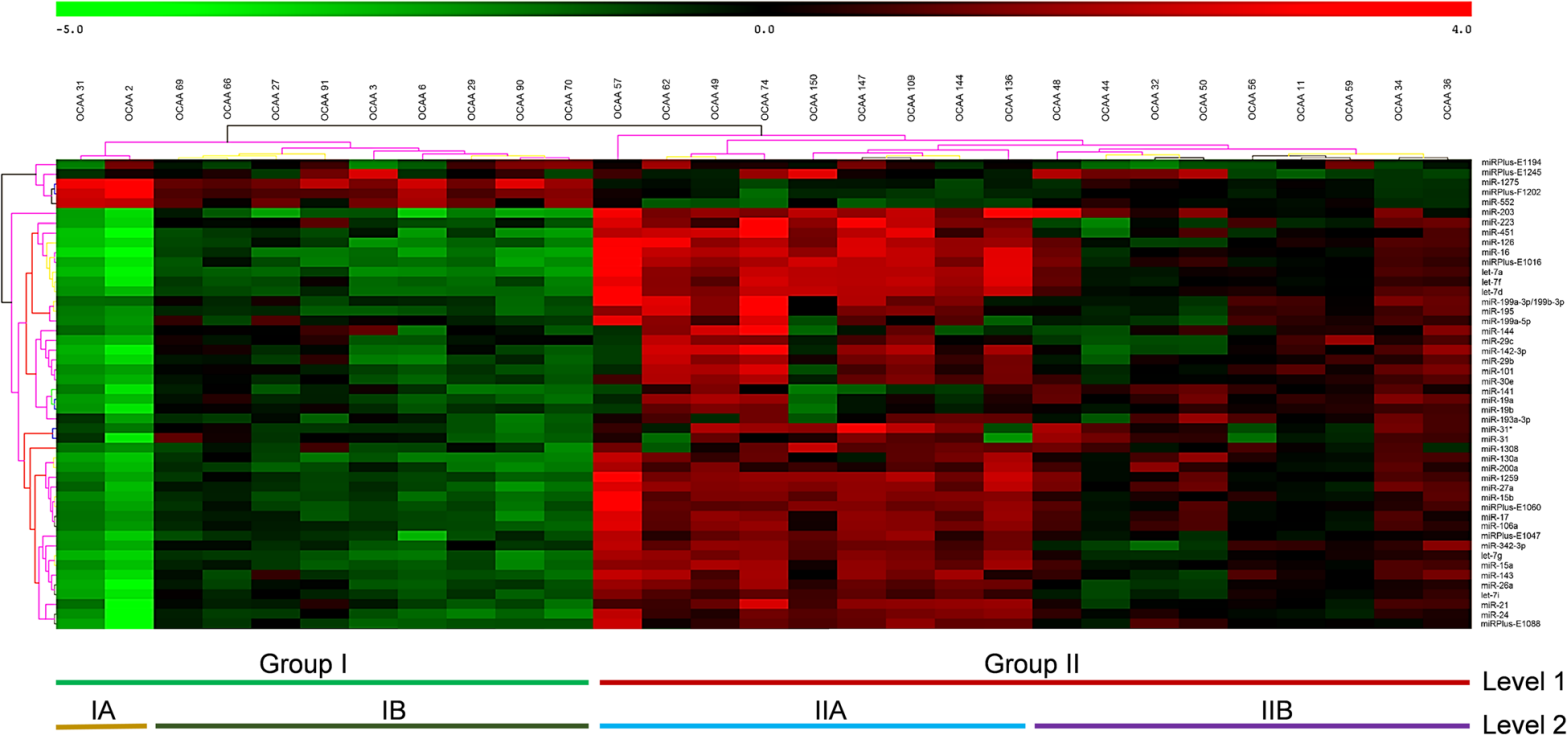
Unsupervised analysis heat map of 29 OSCC primary tumour samples. The clustering is performed on all samples, and on the top 48 microRNAs with highest standard deviation (SD > 1). Each row represents one microRNA and each column represents one sample. The microRNA clustering tree is shown on the left and the sample clustering dendrogram is present on the top. The colour scale shown at the top illustrates the relative expression of a miRNA across all samples: red colour represents an expression level above mean, green colour represents expression level lower than mean. The coloured bars below the heat map represents the grouping of samples based on the branching pattern of the dendrogram. Level I shows the natural clustering of Group I and Group II. Level 2 shows the further sub-grouping of Group I into IA & IB and that of Group II into IIA and IIB

The present study utilized miRCURY LNA™ array (Exiqon, Denmark), that contained capture probes for profiling the expression of 726 mature human miRNAs annotated in miRBase v.16.0 [34], 365 miRNAs proprietary to Exiqon, 77 viral miRNAs, and 18 other small RNAs (snoRNAs, snRNAs and rRNA). Considering the heterogeneity of OSCC, the common reference design [35] was adopted. Profiling was done initially in 21 samples and hence, the common reference pool was composed of these 21 RNA samples. Later, 8 samples were profiled along with one sample from the earlier batch which served as a reference for merging the array data. After image analysis, the obtained data was filtered, normalized and log2-transformed (The microarray data/expression matrix is provided as Additional file 3). The expression of 272 probes were detectable across all the 29 samples and only these miRNAs were included for unsupervised clustering. When sorted by standard deviation (SD), 48 miRNAs had SD > 1 suggesting their differential expression across the samples and the respective heat map is provided as Fig. 1. We then calculated the average ‘delta Log Median Ratio’ (dLMR) values for each of these 48 miRNAs, and classified them as either ‘upregulated’ if the average dLMR value was positive or ‘downregulated’ if the average dLMR value was negative (Table 1). Since the microarray was based on miRBase 16, we verified the MIMATA IDs of the differentially expressed miRNAs except for the Exiqon-proprietary miRNAs, in the current version of miRBase (Release 21, June 2014). Hsa-miR-1259 and hsa-miR-1308 were found to be misclassified dead entries according to miRbase 21, since the former overlapped an annotated snoRNA, while the latter was a fragment of tRNA. Thus, the final list of differentially expressed miRBase annotated miRNAs was limited to 24 downregulated candidates and 15 upregulated ones.

**Table 1:** The top 48 miRNAs identified to be differentially expressed in OSCC by unsupervised clustering analysis of the microarray data

MicroRNA category	Annotated in miRBase v.16 & in v.21 (*n* = 41)	Proprietary to Exiqon (*n* = 7)
Down regulation (*n* = 30) (excluding invalid entries, *n* = 28)	miR-199a/b-3p, miR-15b, miR-16, let-7a, let-7d, miR-126, miR-203, miR-199a-5p, let-7f, miR-17, miR-106a, miR-27a, miR-130a, miR-24, miR-223, miR-143, miR-342-3p, miR-451, let-7g, miR-200a, miR-26a, let-7i, miR-15a, miR-195, *miR-1259*, *miR-1308*	miRPlus-E1016
		miRPlus-E1060
		miRPlus-E1088
		miRPlus-E1047
Up regulation (*n* = 18)	miR-21, miR-30e, miR-31, miR-31*, miR-193a-3p, miR-552, miR-19a, miR-19b, miR-141, miR-101, miR-144, miR-29c, miR-29b, miR-1275, miR-142-3p	miRPlus-E1245
		miRPlus-E1194
		miRPlus-F1202

Italicized and underlined are the misclassified dead entries in miRBase 21

**Fig. 2:**
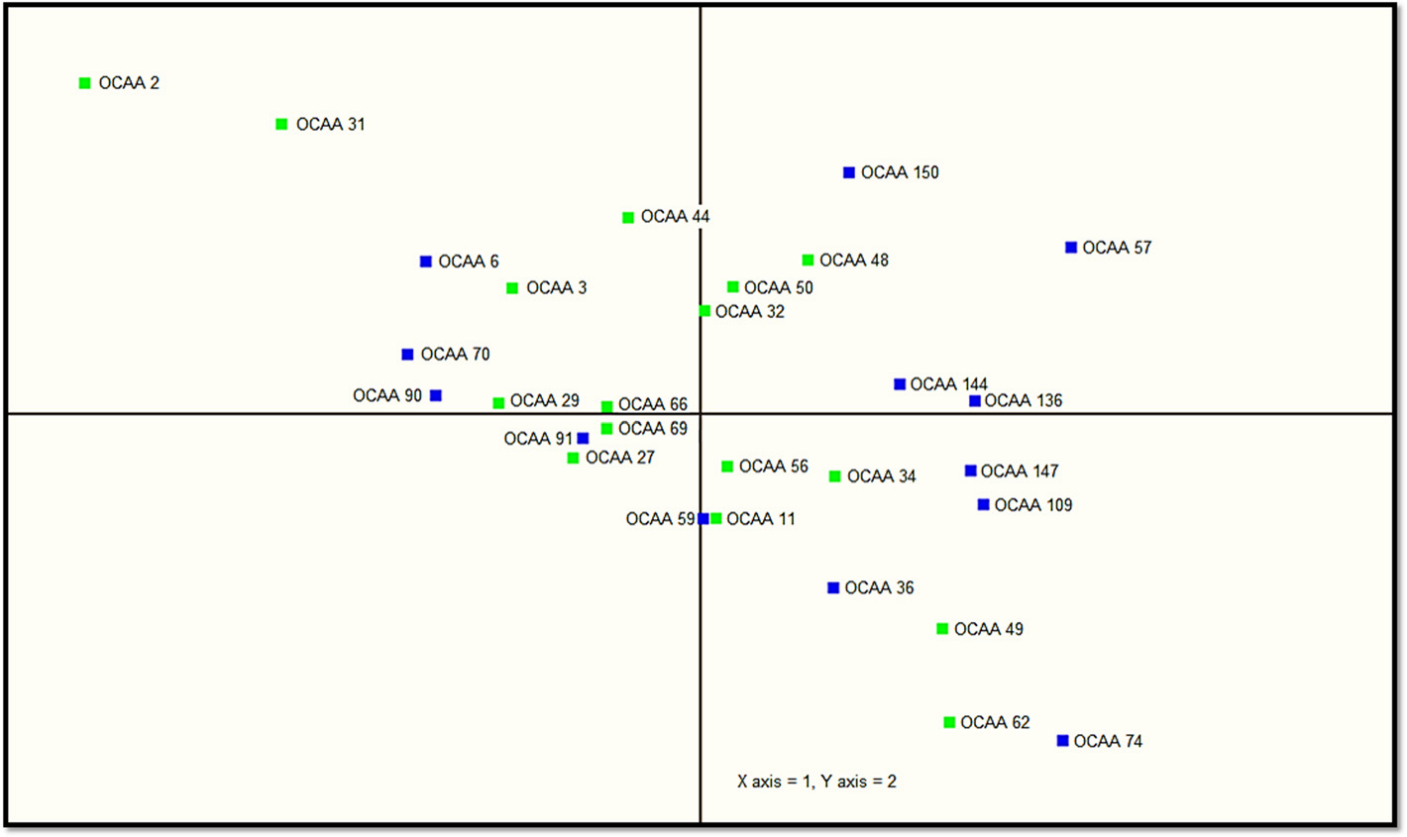
The Principal Component Analysis (PCA) plot of oral cancer samples. The normalized log ratio values have been used for the analysis. OSCC samples of moderately differentiated histology are labeled in green (*n* = 16), and those of well-differentiated histology are labeled in blue (*n* = 13)

In Fig. 1, the sample-clustering tree (dendrogram) on the top indicates the existence of two different molecular groups (Group I and Group II) of the samples based on the differential expression of miRNAs. Therefore, we tested whether Group I and Group II tumours were clustered according to any of their clinico-pathological characteristics (Additional file 4). Surprisingly, no such significant difference was evident. Based on the dendrogram, we further divided Group I and Group II into sub groups (See Fig. 1, lower panel, Level 2 classification) and again tested their association with the clinico-pathological characteristics (Additional file 5). Interestingly, the subgroup IIA and IIB were enriched respectively with tumours of ‘well differentiated’ and ‘moderately differentiated’ histology. The subgroup IB comprised tumours of both histological grades while IA was composed of two tumours obtained from patients of relatively younger age (32 and 45 years). Intrigued by the histological grade difference, this time we performed a supervised clustering analysis by predefining the tumours either as ‘G1 – well differentiated’ or ‘G2 – moderately differentiated’. A two-tailed *T*-test followed by Bonferroni correction [36] implicated that hsa-miR-223, hsa-let-7f and hsa-let-7d could discriminate the G1 and G2 grades of OSCC (Table 2; volcano plot provided as Additional file 6). However, principal component analysis (PCA) showed that the data points were scattered in all quadrants of the plot (Fig. 2) suggesting that neither histopathology nor any other biological component is pronounced in a particular direction. Taken together, our microarray results reiterate the molecular heterogeneity of OSCC.

**Table 2:** MicroRNAs differentially expressed between OSCC samples of moderately differentiated and well-differentiated histology

Annotation	Moderate		Well		Difference	Fold change	p-value
	Average	St.dev	Average	St.dev			
hsa-miR-223	−2.08	1.24	−0.48	1.54	1.59	3.02	<0.006
hsa-let-7f	−2.27	1.31	−0.84	1.54	1.43	2.69	<0.014
hsa-let-7d	−2.54	1.23	−1.35	1.60	1.19	2.28	<0.038

Supervised clustering analysis by predefining the samples into two groups based on their histology, followed by implementation of cut-offs for both significance (*P* < 0.05) and fold change (>2) resulted in the identification of the 3 miRNAs shown in the table above

### RT-qPCR validation of selected differentially expressed miRNAs

To validate the microarray results by reverse transcription quantitative PCR (RT-qPCR) and to identify an appropriate endogenous reference, we primarily assayed the expression of SNORD44 (RNU44), SNORD48 (RNU48), U6snRNA and miR-26b in 10 cancer and 2 control samples. The C_t_ value obtained from the amplification plots (Additional file 7) demonstrated a high consistency in the performance of RNU44 and RNU48, which were subsequently used for normalization. As we have previously confirmed the aberrant expression of two microarray prioritized candidates — miR-21 and miR-143 — in OSCC, as a part of different studies [29, 37], we now evaluated the expression of 10 miRNAs namely hsa-let-7a, hsa-let-7d, hsa-let-7f, hsa-miR-16, hsa-miR-29b, hsa-miR-142-3p, hsa-miR-144, hsa-miR-203, hsa-miR-223 and hsa-miR-1275 using TaqMan® assays in a cohort of 61 OSCC tumours (including the 29 microarray-profiled samples) compared to 9 independent normal oral specimens. A significant upregulation of miR-29b, miR-142-3p, miR-144, miR-203 and miR-223, and a significant downregulation of let-7a, let-7d, let-7f and miR-16, was observed in OSCC compared to controls (Table 3). On the other hand, the levels of miR-1275 were slightly high in tumours with borderline significance.

**Table 3:** The relative expression levels of 10 RT-qPCR validated miRNAs in OSCC samples compared to control adjacent normal tissues

microRNA	Expression level in control samples	Expression level in OSCC samples	*P*-Value
hsa-let-7a	1.00 (0.94–1.45) ^a^	0.46 (0.37–0.58) ^a^	**<0.0001**
hsa-let-7d	1.00 (0.73–1.42) ^a^	0.44 (0.36–0.58) ^a^	**<0.0001**
hsa-let-7f	1.00 (0.86–1.26) ^a^	0.49 (0.41–0.65) ^a^	**<0.0001**
hsa-miR-16	1.00 (0.93–1.09) ^a^	0.66 (0.44–0.92) ^a^	**0.008**
hsa-miR-29b	1.00 (0.87–1.08) ^a^	1.58 (1.02–1.97) ^a^	**0.026**
hsa-miR-142-3p	1.00 (0.56–1.67) ^a^	5.94 (3.26–10.20) ^a^	**<0.0001**
hsa-miR-144	1.67 (0.67–2.67) ^b^	14.26 (3.19–25.34) ^b^	**0.03**
hsa-miR-203	1.00 (0.64–1.58) ^a^	1.82 (0.99–3.57) ^a^	**0.03**
hsa-miR-223	0.97 (0.56–1.38) ^b^	2.67 (2.08–3.26) ^b^	**<0.0001**
hsa-miR-1275	1.05 (0.84–1.25) ^b^	1.33 (1.09–1.57) ^b^	0.06

^a^Median (Interquartile range), since the corresponding expression levels did not follow Gaussian distribution

^b^Mean (95 % confidence limits), since the corresponding expression levels followed Gaussian distribution

Bold faceted are the significant *P*-values (<0.05)

**Fig. 3:**
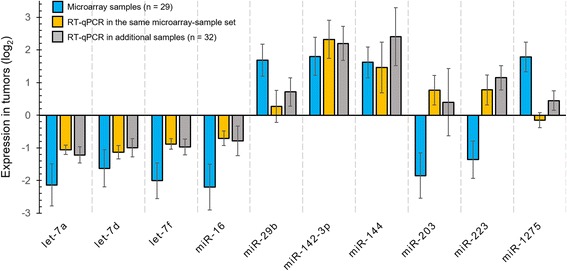
A comparison of the log fold change ratios obtained from microarray and RT-qPCR for the 10 candidate miRNAs. The log2 transformed expression value in OSCC obtained from RT-qPCR for each of the 10 miRNA is compared against its respective delta LogMedianRatio (dLMR) derived from the microarray experiment. For clarity, we separated the 61 OSCC samples into 29 samples that were profiled by microarray and the remaining 32 samples as an additional group. The error bars represent 95 % confidence intervals (CI)

**Fig. 4:**
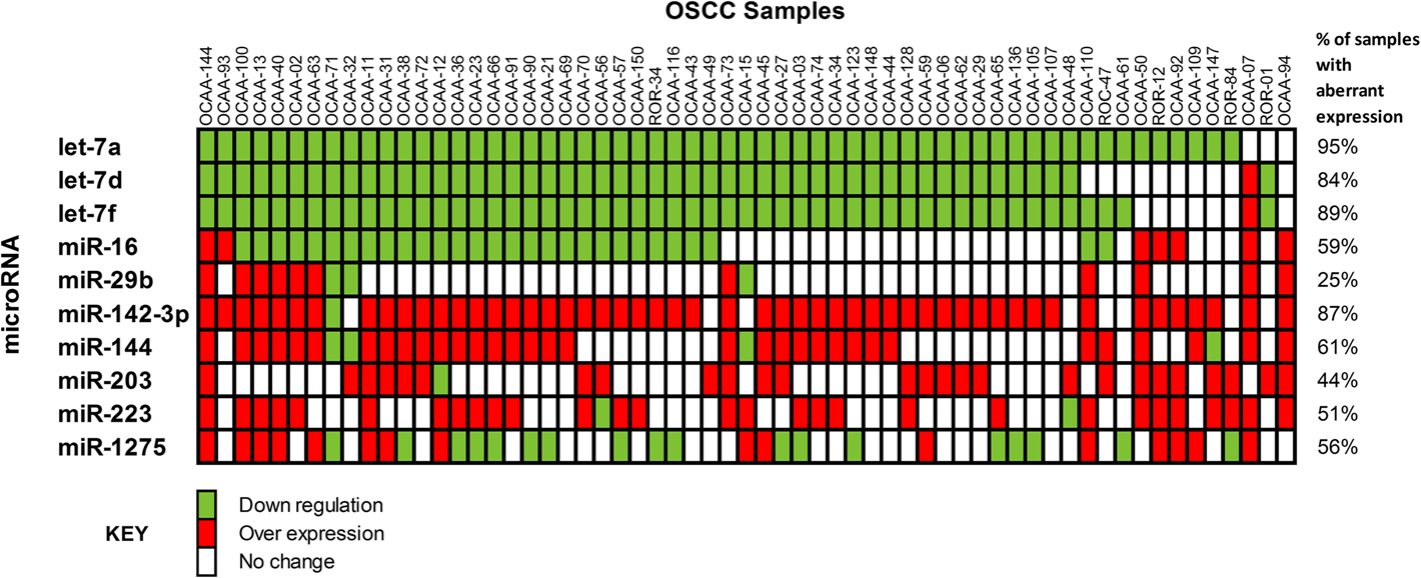
Expression matrix of the 10 RT-qPCR validated miRNAs across the 61 oral squamous cell carcinoma samples. The Expression matrix, otherwise called as the ‘OncoPrint’, represents a graphical summary of alterations in the expression level of the 10 candidate miRNAs as evaluated by RT-qPCR across 61 oral tumours. The colour coded glyphs denote changes in miRNA expression (*red* = overexpression; *green* = down regulation; *white* – expression level within the range observed in the normal control samples). The name of the each miRNA and each sample is provided on the left and top of the matrix, respectively. The values (in %) on the right side of the image and parallel to each miRNA correspond to the total percentage of tumours with altered miRNA expression

For comparison, the log transformed average expression value of each miRNA in tumours as determined by RT-qPCR was plotted against their respective average dLMR values obtained from microarray (Fig. 3). Concordance in up-regulation or down-regulation status amongst the datasets was observed for 7 of the 9 significant miRNAs; opposing expression patterns between microarray and RT-qPCR were seen in case of miR-203 and miR-223. For understanding the extent of alteration of each candidate miRNA across tumours, we constructed an expression matrix using the RT-qPCR data (Fig. 4). Evidently, let-7a, let-7d and let-7f was downregulated across 95, 84 and 89 % of tumours respectively, with a strong tendency for co-occurrence. Downregulation of miR-16 was observed in up to 60 % of the OSCC samples. MiR-142-3p was found to be the top up-regulated miRNA (overexpressed in 87 % percentage of tumours) followed by miR-144 (61 %), miR-223 (51 %), miR-203 (44 %) and miR-29b (25 %). Of note, the level of miR-1275 was aberrant in 56 % of the tumours with downregulation or upregulation seen in equal proportions.

### Association of miRNA expression levels with tumour characteristics

Microarray profiling demonstrated the remarkable heterogeneity in OSCC precluding the identification of a miR signature specifying clinical characteristics. However, individual miRNAs may be influenced by the tumor parameters and association testing in a larger sample size as like our validation cohort may prove fruitful (a summary of the clinical characteristics of the 61 OSCC specimens are presented in Additional file 8). Hence, the tumours were stratified according to their anatomical localization, tumour size, lymph node involvement etc. and tested for any association with the miRNA expression levels (Table 4). The relative expression of let-7d was significantly high in males compared to females, while that of let-7a and let-7f was higher in tongue carcinoma compared to gingivo-buccal tumours. Significantly elevated levels of miR-223 and miR-1275 was seen in the advanced tumour group (III-IV and T3-T4) Vs the less severe group (I-II and T1-T2). A similar high expression of miR-223 was found additionally in G1 grade tumours compared to the G2 grade. It should be noted that miR-223 was the top candidate identified by supervised clustering of the microarray samples according to their histopathology. However the other two miRNAs, let-7d and let-7f, did not show such an association. Further, high level of miR-29b was seen in the T3-T4 group relative to T1-T2 tumour category. Upon classifying the tumours based on their regional lymph node involvement, 6 miRNAs exhibited differential expression: miR-29b, miR-142-3p, miR-144, miR-223 and miR-1275 were elevated significantly in nodal invasion positive (N^+^) tumours compared to nodal invasion negative (N^−^) subgroup, while miR-203 exhibited an opposite trend. Another interesting observation was the lower levels of miR-142-3p in smokers relative to non-smokers. When the significance threshold was corrected for multiple hypotheses testing (*P* < 0.005), only the association of high levels of miR-223 with advanced tumour stage/size and that of miR-1275 with N^+^ tumours remained significant.

**Table 4:** Association of miRNA expression levels with clinical variables

Variable	let-7a	let-7d	let-7f	miR-16	miR-29b	miR-142-3p	miR-144	miR-223	miR-203	miR-1275
Age (in yrs.)										
<60	0.49 ± 0.22	0.44 (0.11–1.25)	0.51 (0.2–1.25)	0.6 (0.01–1.45)	1.58 (0.02–7.79)	5.76 (0.07–39.44)	10.05 ± 14.29	2.54 ± 2.12	1.95 (0–9.25)	1.37 ± 0.99
≥60	0.49 ± 0.17	0.43 (0.26–2.98)	0.49 (0.31–2.20)	0.68 (0.22–2.48)	1.59 (0.21–5.37)	6.24 (0.29–17.81)	19.94 ± 64.44	2.86 ± 2.56	1.72 (0.13–11.01)	1.28 ± 0.84
*P value*	0.92	0.69	0.92	0.74	0.63	0.33	0.45	0.6	0.32	0.72
Gender										
Male	0.54 ± 0.16	0.51 (0.31–2.98)	0.55 (0.22–2.2)	0.68 (0.22–2.48)	1.42 (0.02–7.79)	5.61 (0.07–13.41)	6.79 ± 7.35	2.54 ± 2.19	1.89 (0.13–9.25)	1.15 ± 0.55
Female	0.45 ± 0.21	0.42 (0.11–1.63)	0.45 (0.2–1.25)	0.55 (0.01–1.45)	1.59 (0.25–7.18)	6.51 (0.69–39.44)	19.82 ± 56.45	2.78 ± 2.41	1.73 (0–11.01)	1.47 ± 1.11
*P value*	0.06	0.028	0.08	0.58	0.35	0.07	0.19	0.69	0.41	0.15
Anatomical site										
GBC	0.47 ± 0.20	0.42 (0.11–2.98)	0.46 (0.2–2.2)	0.65 (0.01–2.48)	1.56 (0.02–7.79)	6.33 (0.07–39.44)	16.03 ± 47.12	2.74 ± 2.16	1.72 (0–11.01)	1.34 ± 0.98
Tongue	0.60 ± 0.14	0.52 (0.35–0.76)	0.59 (0.47–0.92)	0.66 (0.42–1.32)	1.63 (0.54–2.44)	4.36 (0.48–9.37)	5.28 ± 5.06	2.31 ± 3.05	2.69 (0.36–4.3)	1.28 ± 0.61
*P value*	0.05	0.16	0.01	0.85	0.99	0.09	0.12	0.59	0.25	0.84
Clinical stage										
I or II	0.60 ± 0.20	0.55 (0.34–0.82)	0.67 (0.43–0.78)	0.77 (0.4–1.11)	0.84 (0.58–1.61)	2.17 (0.48–9.37)	2.80 ± 2.39	1.17 ± 0.63	3.14 (1.73–6.14)	0.92 ± 0.26
III or IV	0.49 ± 0.20	0.44 (0.23–2.98)	0.51 (0.22–2.2)	0.6 (0.01–2.48)	1.61 (0.02–7.79)	5.94 (0.07–39.44)	17.11 ± 48.91	2.84 ± 2.49	1.82 (0.26–9.25)	1.40 ± 1.01
*P value*	0.33	0.63	0.39	0.68	0.06	0.1	0.05	**0.004**	0.17	0.03
Tumour Size										
T1 or T2	0.56 ± 0.16	0.44 (0.34–0.82)	0.58 (0.42–0.78)	0.79 (0.4–1.13)	1.01 (0.54–1.70)	2.93 (0.48–15.76)	3.56 ± 3.52	0.95 ± 0.62	2.73 (0.53–7.9)	1.02 ± 0.28
T3 or T4	0.49 ± 0.21	0.46 (0.23–2.98)	0.49 (0.22–2.2)	0.55 (0.01–2.48)	1.67 (0.02–7.79)	5.94 (0.07–39.44)	19.02 ± 52.15	3.14 ± 2.53	1.75 (0.26–9.25)	1.44 ± 1.07
*P value*	0.31	0.91	0.23	0.13	0.006	0.21	0.07	**≤** **0.0001**	0.11	0.03
Nodal invasion										
N-	0.60 ± 0.18	0.55 (0.34–0.82)	0.67 (0.43–0.98)	0.64 (0.4–1.11)	0.84 (0.45–1.61)	1.84 (0.48–9.37)	2.20 ± 2.09	1.48 ± 0.85	4.24 (1.73–9.25)	0.85 ± 0.23
N+	0.49 ± 0.20	0.44 (0.23–2–98)	0.51 (0.22–2.2)	0.6 (0.01–2.48)	1.65 (0.02–7.79)	6.15 (0.07–39.44)	17.82 ± 49.88	2.87 ± 2.54	1.75 (0.26–7.9)	1.43 ± 1.02
*P value*	0.22	0.63	0.31	0.78	0.009	0.007	0.04	0.01	0.01	**0.003**
Histological grade										
G1/well	0.53 ± 0.25	0.43 (0.23–1.63)	0.46 (0.22–1.25)	0.67 (0.22–1.45)	1.59 (0.02–7.79)	7.52 (0.07–39.44)	21.97 ± 68.38	3.42 ± 2.95	2.21 (0.26–11.01)	1.43 ± 0.88
G2/moderate	0.47 ± 0.13	0.44 (0.26–0.85)	0.52 (0.34–0.98)	0.57 (0.01–1.13)	1.60 (0.45–7.18)	5.28 (0.67–15.76)	9.79 ± 14.40	1.99 ± 1.33	1.74 (0.28–9.25)	1.18 ± 0.97
*P value*	0.34	0.78	0.96	0.23	0.77	0.25	0.41	0.04	0.42	0.33
Smoking										
Yes	0.56 ± 18	0.46 (0.32–2.98)	0.55 (0.22–2.2)	0.68 (0.22–2.48)	1.23 (0.02–4.92)	3.6 (0.07–13.41)	4.72 ± 4.76	2.62 ± 2.54	1.96 (0.27–9.25)	1.06 ± 0.59
No	0.48 ± 0.20	0.44 (0.23–1.63)	0.47 (0.27–1.25)	0.59 (0.01–1.45)	1.64 (0.25–7.79)	6.27 (0.69–39.44)	18.90 ± 52.86	2.73 ± 2.38	1.84 (0.26–7.90)	1.43 ± 1.05
*P value*	0.21	0.43	0.12	0.62	0.07	0.03	0.1	0.88	0.72	0.11
Tobacco chewing										
Yes	0.47 ± 0.17	0.44 (0.23–2.98)	0.46 (0.27–2.2)	0.65 (0.01–2.48)	1.61 (0.25–7.18)	5.94 (0.69–17.81)	19.41 ± 54.98	2.89 ± 2.65	1.72 (0.26–7.9)	1.46 ± 1.07
No	0.55 ± 0.25	0.43 (0.26–1.25)	0.55 (0.22–1.25)	0.66 (0.22–1.45)	1.25 (0.02–7.79)	4.32 (0.07–39.44)	6.10 ± 5.51	2.31 ± 1.76	3.44 (0.36–9.25)	1.04 ± 0.62
*P value*	0.22	0.74	0.24	0.66	0.27	0.13	0.15	0.42	0.06	0.07
Alcohol intake										
Yes	0.52 ± 0.16	0.57 (0.32–2.98)	0.53 (0.22–2.2)	0.66 (0.22–2.48)	1.65 (0.02–4.92)	5.46 (0.07–13.41)	7.15 ± 7.41	2.97 ± 2.59	1.75 (0.27–5.97)	1.17 ± 0.61
No	0.49 ± 0.21	0.42 (0.23–1.63)	0.47 (0.27–1.25)	0.65 (0.01–1.45)	1.56 (0.25–7.79)	6.38 (0.48–39.44)	18.32 ± 53.62	2.60 ± 2.35	2.09 (0.26–9.25)	1.39 ± 1.07
*P value*	0.55	0.06	0.38	0.92	0.94	0.3	0.21	0.62	0.54	0.34

For miRNAs that followed Gaussian distribution, the expression levels are provided as mean ± SD. For miRNAs that did not follow Gaussian distribution, the expression levels are provided as median (range). The *P*-Value for significance was adjusted for multiple hypothesis testing to *P* = 0.05/10 = 0.005. Thus a *P*-value between 0.05 and 0.005 should be regarded as borderline significance. GBC, gingivo-buccal complex

### Integrating miRNAs to tumourigenesis and functional pathways

**Fig. 5:**
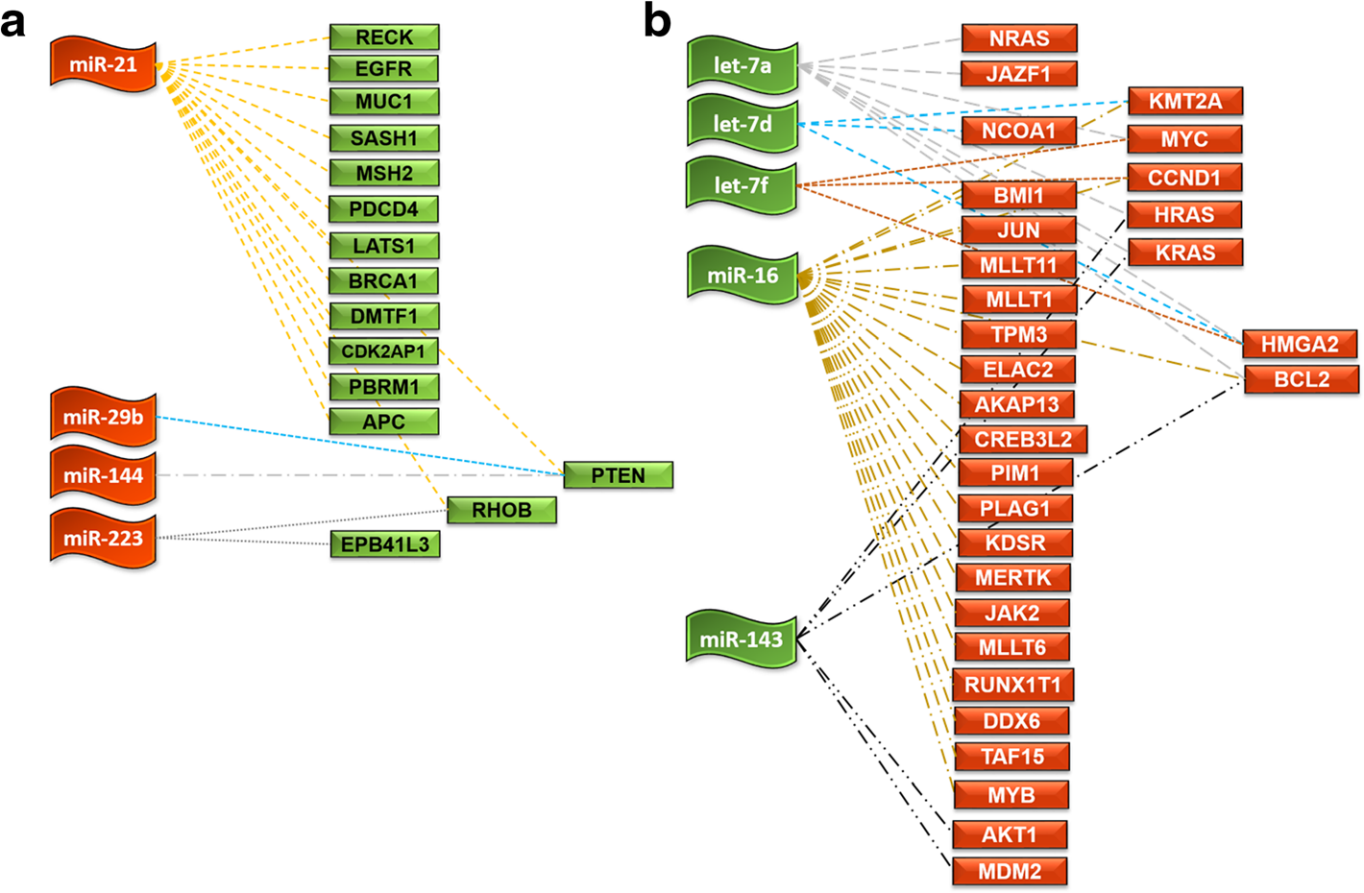
Inverse relationship of upregulated miRs with oncogenes, and downregulated miRs with tumour suppressor genes. Shown here are the interactions between (**a**) the over expressed miRNAs and tumour suppressor genes, and (**b**) the down regulated miRNAs and oncogenes. Data obtained by comparing the validated targets of each miRNA obtained from miRTarBase against the list of manually curated ‘Tumour suppressor genes’ or ‘Oncogenes’ retrieved from UniProt Knowledgebase. The *flags* represent miRNAs, the *rectangular boxes* represent their target genes and the *discontinuous lines* connecting them represent the interaction

Because miRNAs function through post-transcriptional gene repression, from miRTarBase [30] we retrieved (August, 2015) the experimentally validated target genes of hsa-let-7a, hsa-let-7d, hsa-let-7f, hsa-miR-16, hsa-miR-29b, hsa-miR-142-3p, hsa-miR-144, hsa-miR-203, and hsa-miR-223 that showed significant differential expression in the present study together with miR-21 and miR-143 that were previously validated to be deregulated in OSCC by us [29, 37]. Altogether, these 11 miRNAs cumulatively targeted 2121 genes, of which 366 were targeted by multiple miRNAs (ranging from 2 to 6) thus raising the total number of miRNA:mRNA interactions to 2388 (Additional file 9). To gain a specific insight in cancer context, we parsed the target genes of upregulated or downregulated miRNAs and compared them against the list of manually curated ‘tumour suppressor genes’ or ‘oncogenes’ respectively (see Methods section for details). This analysis corroborated the inverse relationship of oncomiRs with tumour suppressor genes and of tumour suppressor miRs with oncogenes (Fig. 5). As anticipated, miR-21 targeted the largest number of tumour suppressors (*n* = 14), among which *PTEN* was additionally targeted by miR-29b and miR-144, while *RHOB* was also repressed by miR-223. On the other hand, miR-16 was the most versatile miRNA targeting 21 oncogenes in total. Some of the notable oncogenes that had multiple miRNA interactions were *BCL2* (let-7a, miR-16, and miR-143), *HMGA2* (let-7a, let-7d, and let-7f), *CCND1* (let-7f, miR-16), *MYC* (let-7a, let-7f), *HRAS* and *KRAS* (let-7a, miR-143), and KMT2A (let-d, miR-16).

**Fig. 6:**
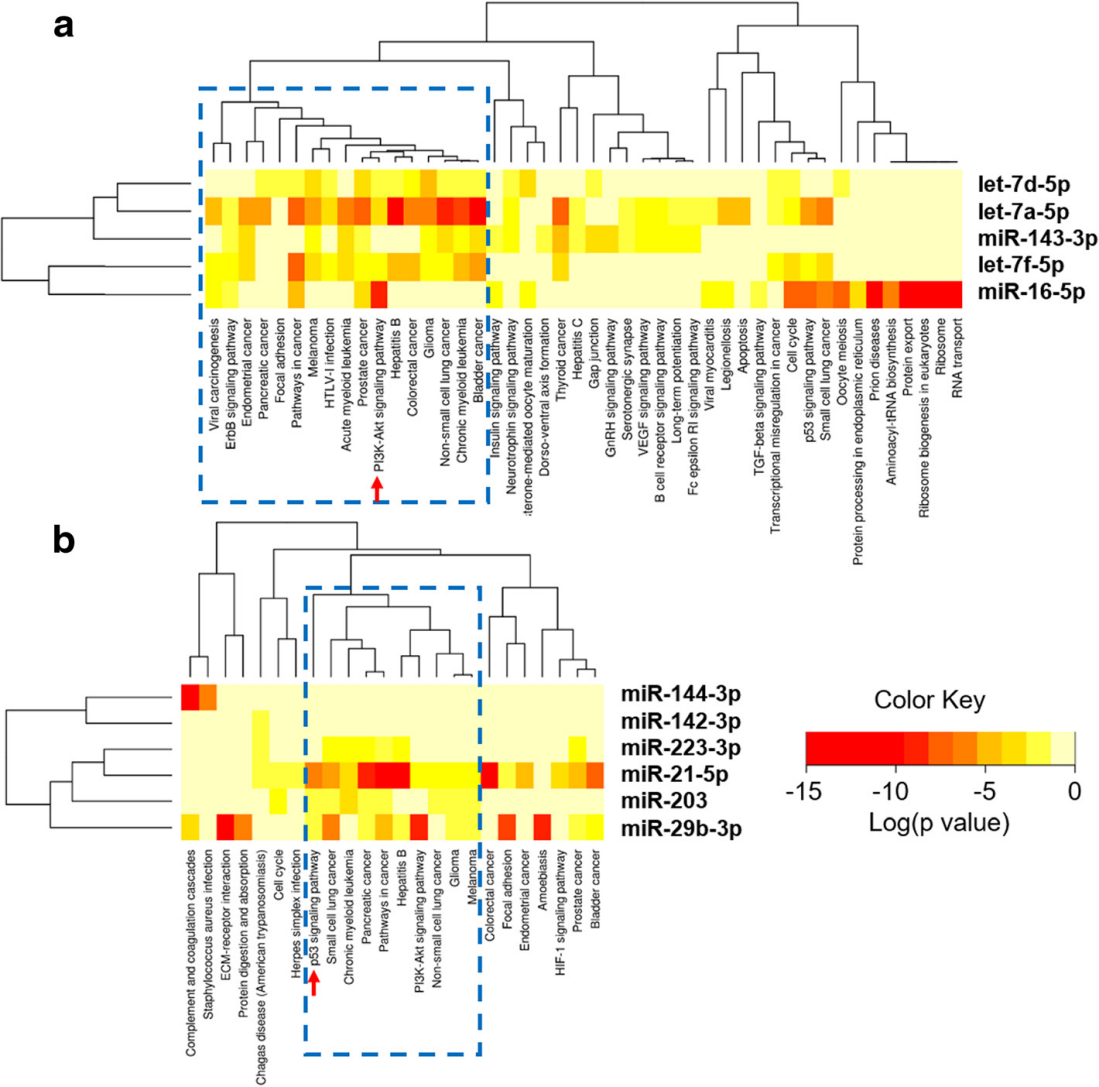
Heat map of differentially expressed miRNAs versus significantly enriched functional pathways. **a** heat map of downregulated miRNAs Vs functional pathways; **b** heat map of upregulated miRNAs Vs functional pathways. In both heat maps, darker colours represent higher statistical significance as indicated by the colour key at the bottom. The attached dendrograms on both axes depict hierarchical clustering results for miRNAs and pathways, respectively. Boxed by dashed lines are the functionally relevant pathway clusters that overlap the highest number of miRNAs. Indicated by arrow mark are the pathways that were explored further. Figure developed from the output of Diana miRpath V.2

**Fig. 7:**
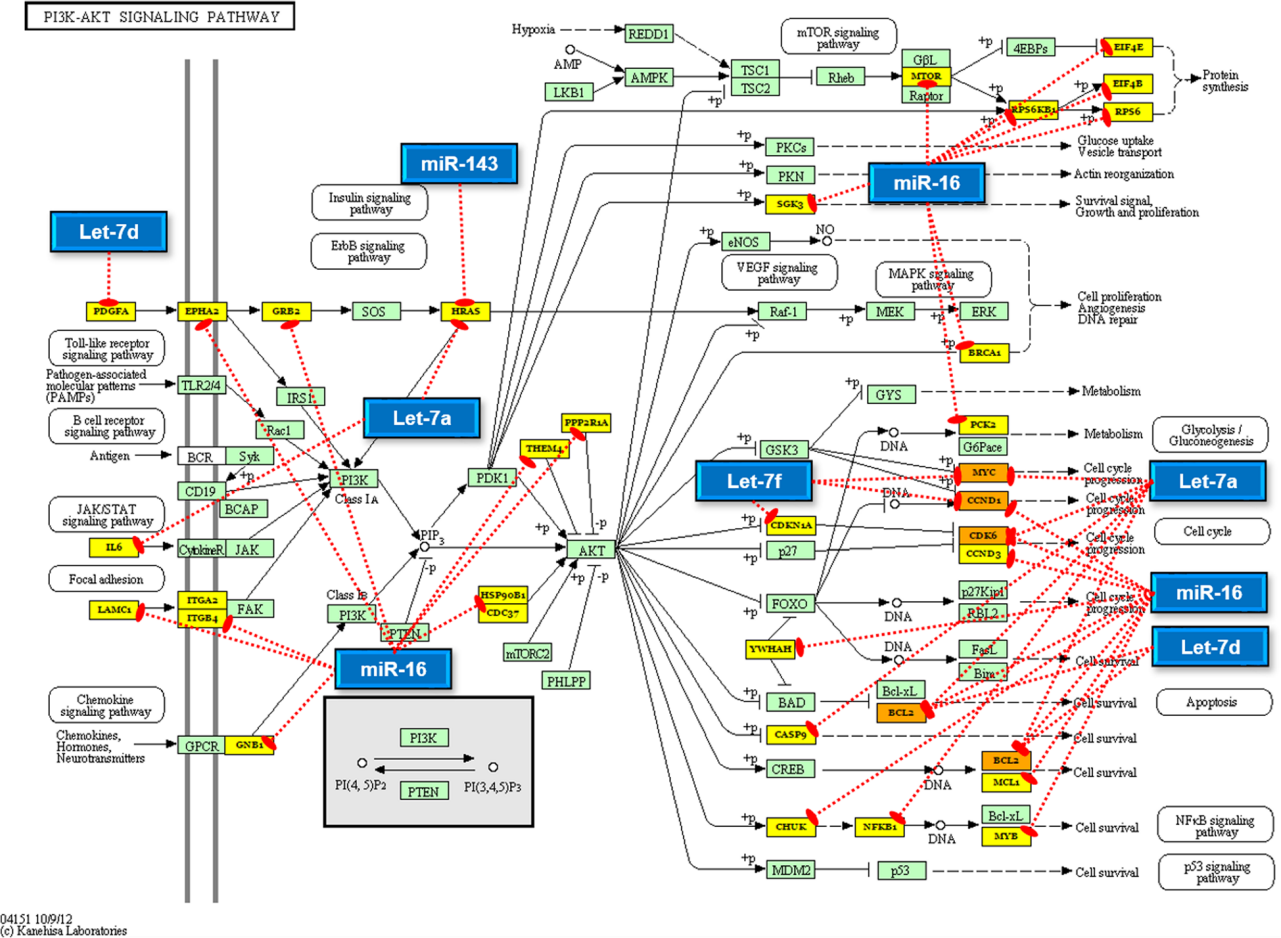
MicroRNAs downregulated in OSCC target multiple genes in the PI3K/AKT pathway. Shown here are the 5 miRNAs significantly downregulated in OSCC and their validated target genes that participate in the PI3K/Akt signaling pathway (hsa04151). *Blue* rectangles represent the miRNAs, *yellow* rectangles represent genes targeted by only one miRNA, and *orange* rectangles represent genes targeted by more than one miRNA. *Green* rectangles represent genes that are not targeted by any of the miRNAs studied herein. Figure developed from the output of Diana miRpath V.2

**Fig. 8:**
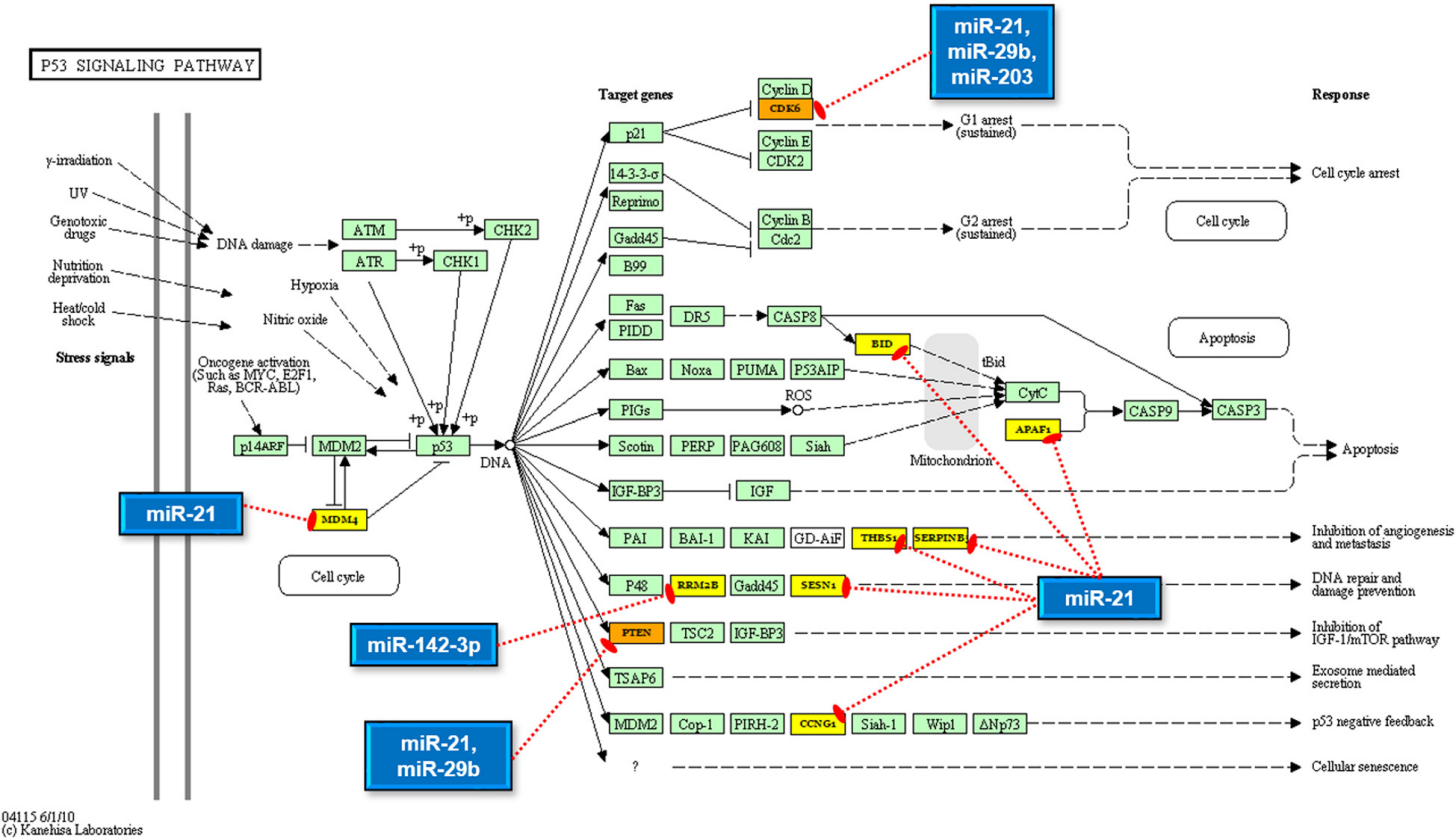
MicroRNAs upregulated in OSCC repress multiple genes in the p53 signaling pathway. Shown here are the 4 miRNAs significantly upregulated in OSCC and their validated target genes that participate in the p53 signaling pathway (hsa04115). *Blue* rectangles represent the miRNAs, *yellow* rectangles represent genes targeted by only one miRNA, and *orange* rectangles represent genes targeted by more than one miRNA. *Green* rectangles represent genes that are not targeted by any miRNA studied herein. Figure developed from the output of Diana miRpath V.2

Further, we separately examined the targets of downregulated and upregulated miRNAs, for enriched functional pathways using DIANA-miRPath v.2 [33]. The miRNA vs pathway heatmaps provided in Fig. 6 show that ‘pathways in cancer’, ‘prostate cancer’, ‘non-small cell lung cancer’, ‘glioma’, ‘pancreatic cancer’, ‘chronic myeloid leukaemia’ etc., were significantly enriched in case of both downregulated and upregulated miRNAs. As this observation is accountable to the functional redundancy of genes across multiple cancer types, we focused our attention on to specific signaling mechanisms. Remarkably, 48 genes at different levels of the PI3K/Akt signaling pathway were targeted by the tumour suppressor miRs let-7a, let-7d, let-7f, miR-16, and/or miR-143 (Fig. 7) whereas 11 genes of the p53 signaling pathway were targeted by the oncomiRs miR-21, miR-29b, miR-142-3p and miR-203 (Fig. 8). Based on these results, we propose that the aberrantly expressed miRNAs may simultaneously elicit the constitutive activation of PI3K/Akt pathway and the suppression of the p53 pathway.

## Discussion

OSCC is as a major life-threatening malignancy in India [3, 38]. Geographical differences in incidence and mortality, heterogeneity in anatomical localization, association with a broad spectrum of risk factors, and the unpredictable treatment outcome renders OSCC as one of the most complex cancer. Despite multimodal therapy, the 5-year survival rates of OSCC patients has not crossed 50 % for the past 4 decades [3, 7]. Accumulating evidences suggest that miRNAs may not only prove useful as diagnostic and prognostic markers for cancer, but also offer the competency for targeted therapies [39]. Hence, microarray and RT-qPCR has been widely applied by various researchers to identify miRNAs that are differentially expressed in head and neck/oral cavity cancers [18–23, 40–42]. However, there has been a difficulty in characterizing a consistent miRNA signature. Also, because of the constant discovery of new mature miRNAs, the expression of several of them remains to be studied. Hence, evaluating global miRNA expression and testing their association with tumour characteristics is a promising requisite to improve diagnosis, approaches to therapy and to reduce the burden of OSCC in India.

In the current study, the expression of 1,168 miRNAs were profiled in 29 OSCC primary tissues of south Indian ethnicity using miRCURY LNA™ array that offers key benefits such as high specificity, high sensitivity, and the efficient discrimination of closely related miRNA species [43]. Moreover, the common reference design adopted herein is considered to be advantageous as it ideally contains all miRNAs found in any of the samples, offers direct comparison and normalization of individual samples to a common factor, allows the separation of technical and biological variation, and the identification of outlying samples among replicates [35]. Further, the common reference is ideal for heterogeneous samples and when the profiling is performed in batches [35]. Unsupervised hierarchical clustering of the tumours and miRNAs suggested that the naturally arising clusters do not represent the clinical tumour characteristics. Principle component analysis indicated that the miRNA expression is not pronounced in any specific direction further confirming the molecular heterogeneity of OSCC. A similar molecular heterogeneity in Indian OSCC unrelated to differentiation subtypes or clinical tumour stage was reported already by a study when a clustering analysis of samples was performed with differentially expressed miRNAs [44]. This suggests the need for more studies with large number of samples to advocate a ‘miRNA expression-based classification’ system of OSCC and to test the association of different classes with prognosis. Out of the 46 differentially expressed miRNAs identified by microarray, we validated the expression of 10 miRNAs by RT-qPCR in a cohort of 61 OSCC samples compared to 9 independent normal oral tissues. While we faced some complications in obtaining paired normal tissues, previous studies have demonstrated that control tissues amounting to 10 % of the total sample size is adequate [45, 46]. A general agreement in significance and the trend of expression was observed between microarray and RT-qPCR for let-7a, let-7d, let-7f, miR-16, miR-29b, miR-142-3p, and miR-144. Although both microarray and RT-qPCR confirmed the differential expression of miR-203 and miR-223 in OSCC, a difference in the expression pattern was observed between both techniques. The expression of miR-1275 significantly varied only between nodal invasion positive and nodal invasion negative tumours but was near borderline significance when all tumours were considered as a single group and compared against the controls. Discrepancies or low correlation in expression profiles between the array and RT-qPCR is not new as it has been previously reported and suggested to have arisen due to the decreased sensitivity, reliability and a higher false positive rate associated with microarray [47, 48].

Let-7 miRNAs regulate the expression of *RAS* [49] and several other genes involved in the cell cycle thereby repressing cell division both directly and indirectly [50]. Reduced expression of most members of the let-7 family has been noted as a characteristic feature of HNSCC [17, 51]. It is interesting to note that *RAS* oncogene activation by mutation and amplification was reported to be more frequent (>25 %) in OSCC of India [52–54]. Even the precursor molecules of let-7a and let-7d were reported to be down regulated in HNSCC and additionally, low levels of let-7d had an association with poorer prognosis [20]. Recently, let-7a was found to repress stemness-associated genes in tumour-initiating cells [55] and let-7d was shown to negatively modulate EMT in OSCC cell lines [56]. Similar to let-7, miR-16 is frequently deleted and/or down regulated in many types of cancer [57]. Several targets of miR-16 including *BCL2*, *MCL1*, *CCND1* and *WNT3A* may explain its role in modulating the cell cycle, inhibiting cell proliferation, promoting apoptosis and suppressing tumourigenicity both in vitro and in vivo [57, 58]. However, miR-16 was reported to be upregulated in OSCC cell lines [17] and tumour tissues [21], whereas it was downregulated in tumours of the OSCC animal model [59]. Notably, a study on Indian OSCC samples showed that a decrease in the expression of miR-16 along with miR-125a and miR-184 is associated with oral tumourigenesis [60]. Supporting their general tumour suppressive nature, we observed the downregulation of let-7a, let-7b, let-7d, and miR-16 in OSCC. Moreover, the difference in the levels of let-7d between males and females, and that of let-7a and let-7f between tumours of the tongue and gingivo-buccal complex certainly needs confirmation.

Herein, miR-29b was overexpressed in OSCC, a phenomenon also seen in HNSCC tissues and cell lines [17, 21]. On the contrary, enforced expression of miR-29 family inhibited tumourigenicity in lung cancer cell lines [61], suggesting their reversed roles in different types of cancer. miR-142-3p was first identified for its function in the development of the lymphoid system [62], and was subsequently implicated as an oncomiR in leukaemia [63]. Deregulation of miR-142-3p and miR-142-5p along with 33 other miRNAs constituted a miR signature specific to malignant oral neoplasms [46]. Our results on the upregulation of miR-142-3p in OSCC agrees well with previous HNSCC studies [19, 21]. Although miR-144 was found to be frequently upregulated in nasopharyngeal carcinoma [64], its differential expression in OSCC has not been previously described. We noticed a > 8 fold upregulation of miR-144 in OSCC compared to controls. The association of miR-29b with tumour size, that of miR-29b, miR-142-3p and miR-144 with regional lymph node invasion, and the additional link between miR-142-3p and smoking clearly warrants further confirmation from upcoming studies.

miR-203 is a stemness-inhibiting miRNA that induces epidermal differentiation by targeting ΔNp63 and restricting proliferative potential [65, 66]. In addition, genotoxic damage in JHU-012 (HNSCC) cell line simultaneously increased miR-203 expression and decreased ΔNp63 levels thereby inducing cell death [65]. In oral cancer, miR-203 was observed to be downregulated due to DNA hypermethylation [16]. In our study, the average dLMR value obtained from microarray indicated that miR-203 is generally downregulated in OSCC, while RT-qPCR demonstrated its overexpression. This discordance may be attributed to (i) the use of different calibrator for normalization (common reference pool in microarray and normal tissues in RT-qPCR), (ii) different sample size, (iii) different time points at which these experiments were performed, and (iv) the use of different quantitative values for expression (dLMR for microarray and fold change in case of RT-qPCR). However, the decreased levels of miR-203 in N^+^ tumours highlight its anti-proliferative and anti-metastatic potential. As like miR-203, the expression of miR-223 also showed different trends when assessed by microarray and RT-qPCR. Nonetheless, the level of miR-223 was significantly higher in tumours of advanced stage/size even after Bonferroni correction, implying its participation in tumour growth and invasion. Enhanced expression of the hematopoietic specific [67] miR-223 has been consistently observed in HNSCC [21, 68]. Recent studies demonstrate the over expression of miR-1275 in adrenocortical carcinoma patients [69]. Moreover, high levels of miR-1275 were also found in breast cancer cell lines and prostate cancer cell line whereas low levels was characteristic of a glioma cell line [70]. Dysregulation of miR-1275 has not been reported until now in OSCC/HNSCC and our results reveal high levels of this particular miRNA in N^+^ tumours as compared to N^−^ tumors, an observation that was significant even after Bonferroni correction. Further research in this direction will aid in elucidating the benefits of using some of these miRNAs as therapeutic targets in the battle against cancer.

Despite the association of several miRNAs with OSCC/HNSCC, little is known about the functional pathways. Numerous studies have identified several target genes for each miRNA and hence we investigated the experimentally confirmed targets of the RT-qPCR validated miRNAs for enriched functions and pathways. Construction and visualization of a miRNA:mRNA interaction network suggested the involvement of 2121 genes. Considering that the human genome contains ~ 30,000 protein coding genes, there is a possibility of up to 7.07 % of them being deregulated by the cumulatively action of let-7a, let-7d, let-7f, miR-16, miR-29b, miR-142-3p, miR-144, miR-203, miR-223, miR-21 and miR-143. Comparison of the targets of downregulated miRNAs against ‘Oncogenes’ and that of upregulated miRNAs against ‘tumour suppressor genes’ demonstrated the existence of an inverse relationship. We also observed a strong link between the downregulated miRNAs and the PI3K/Akt signalling pathway. Accumulating genetic and cancer biology evidence demonstrate that PI3K/AKT pathway is under the tight regulation of miRNAs [71, 72]. The PI3K/PTEN/AKT/mTOR signaling axis is critical for maintaining the homeostasis in proliferation, metabolism, migration, apoptosis, etc., [73] and its deregulation or constitutive activation due to mutations has been implicated in oral carcinogenesis [74–76]. More than 47 % of HNSCC and specifically 38 % of Indian OSCC samples have been suggested to carry at least one molecular alteration in this pathway [74, 77]. Thus, loss of miRNAs’ control on PI3K/AKT signaling can have dire biological consequences. Alternatively, the overexpression of miR-21 and miR-29b can repress PTEN preventing it from negatively regulating the PI3K/AKT signaling.

A similar enrichment analysis of the target genes of upregulated miRNAs revealed that P53 signaling pathway is commonly affected. The pivotal role of p53 in tumour suppression is evident from the fact that more than 50 % of HNSCCs harbour inactivating p53 mutations or loss of its genomic loci [74, 78, 79]. Mutations apart, p53 is controlled at several levels including transcription, post-transcriptional regulation by miRNAs, post-translational modification etc. Several miRNAs have been shown to directly target and impair p53 functions, while there are also miRNAs regulated by the transcriptional activity of p53 [80]. Since p53 mutations are relatively infrequent in the Indian OSCC population compared to the world [81–83], we propose that miRNA mediated repression may operate as an alternate mechanism of p53 inactivation. In summary, aberrant expression and dysfunction of miRNAs may be reckoned as a crucial step in oral cancer initiation and progression. Some of the presently studied miRNAs may turn out to be important diagnostic, prognostic and predictive biomarkers for OSCC in the future.

## Conclusion

Research striving towards early detection and efficient management of OSCC is of utmost importance in India, which is considered as the world capital of oral cancer. As evidences point out the benefits and practicality of using miRNAs as cancer biomarkers, we profiled the expression of 1168 microRNAs (miRNAs) in primary OSCC tumours by microarray. Unsupervised analysis identified 46 miRNAs to be differentially expressed in OSCC. Clustering of the samples based on the dendrogram and principle component analysis demonstrated the high heterogeneity of OSCC. Validation of 10 differentially expressed candidates was carried out using mature miRNA specific stem-loop primers and TaqMan probes in a spectrum of 61 OSCC samples compared to 9 independent adjacent normal tissues. The reduced expression of let-7a, let-7d, let-7f, miR-16, and overexpression of miR-29b, miR-142-3p, miR-144, miR-203, and miR-223 were confirmed in OSCC, while miR-1275 showed borderline statistical significance. Univariate analysis on samples stratified according to their tumor characteristics followed by Bonferroni correction indicated that high levels of miR-223 was associated with advanced tumor stage and size whereas high levels of miR-1275 were associated with regional lymph node invasion. To comprehend the signaling pathways and functional networks participating in OSCC pathogenesis, the validated targets of the significantly deregulated miRNAs were retrieved from miRTarBase database and subjected to KEGG pathway enrichment analyses. The downregulated miRNAs targeted genes enriched in the PI3K/Akt signaling thereby activating limitless replication potential. Conversely, the up regulated miRNAs targeted genes enriched in the p53 signaling pathway, the inhibition of which may lead to evasion of apoptosis and defects in cell cycle checkpoints. Taken together, the results suggest that alterations in the cellular level of miRNAs might have a major impact on the proteome of the cancer cell thereby contributing to oral tumorigenesis.

## Additional files

Additional file 1: Representative Bioanalyzer 2100 electropherogram of a good quality, and fully degraded RNA. (DOCX 233 kb) Additional file 2: Details of miRNA assays used in the study. (DOCX 14 kb) Additional file 3: Microarray data providing the expression matrix and normalization values for samples of Batch I, Batch II, Batch I and II merged and the outcome of unsupervised and supervised clustering analysis. (XLSX 2362 kb) Additional file 4: Association testing of the 2 major clusters of OSCC samples with their clinico-pathological characteristics. (DOCX 15 kb) Additional file 5: Clinico-pathological characteristics of the 4 subgroups of oral cancer samples based on unsupervised clustering of microarray data. (DOCX 14 kb) Additional file 6: Volcano plot of the supervised clustering analysis of microarray data. (DOCX 92 kb) Additional file 7: Box and whisker plot of the C_t_ values and their corresponding amplification plot of the 4 reference small RNAs. (DOCX 314 kb) Additional file 8: Consolidated clinico-pathological profile of the 61 OSCC samples included in RT-qPCR validation. (DOCX 14 kb) Additional file 9: CytoScape representation of the miRNA:mRNA interaction network. (DOCX 1528 kb)

## Abbreviations

3’-UTR: 3’-untranslated region
GBC: gingivo-buccal complex
HNSCC: head and neck squamous cell carcinoma
miRNA: microRNA
mRNA: messenger RNA
OSCC: oral squamous cell carcinoma
RNA: ribonucleic acid
RT-qPCR: reverse transcriptase quantitative polymerase chain reaction
