# Supplementary material for: Oral squamous cell carcinoma: microRNA expression profiling and integrative analyses for elucidation of tumourigenesis mechanism
Source: Mol Cancer. 2016 Apr 7;15:28. doi: 10.1186/s12943-016-0512-8 (PMC4823852; doi:10.1186/s12943-016-0512-8)
Supplement: Additional file 1: — Representative Bioanalyzer 2100 electropherogram of a good quality, and fully degraded RNA. (DOCX 233 kb) [file 12943_2016_512_MOESM1_ESM.docx]

**Additional File 1: Representative Bioanalyzer 2100 electropherogram of a good quality, and fully degraded RNA.**

**
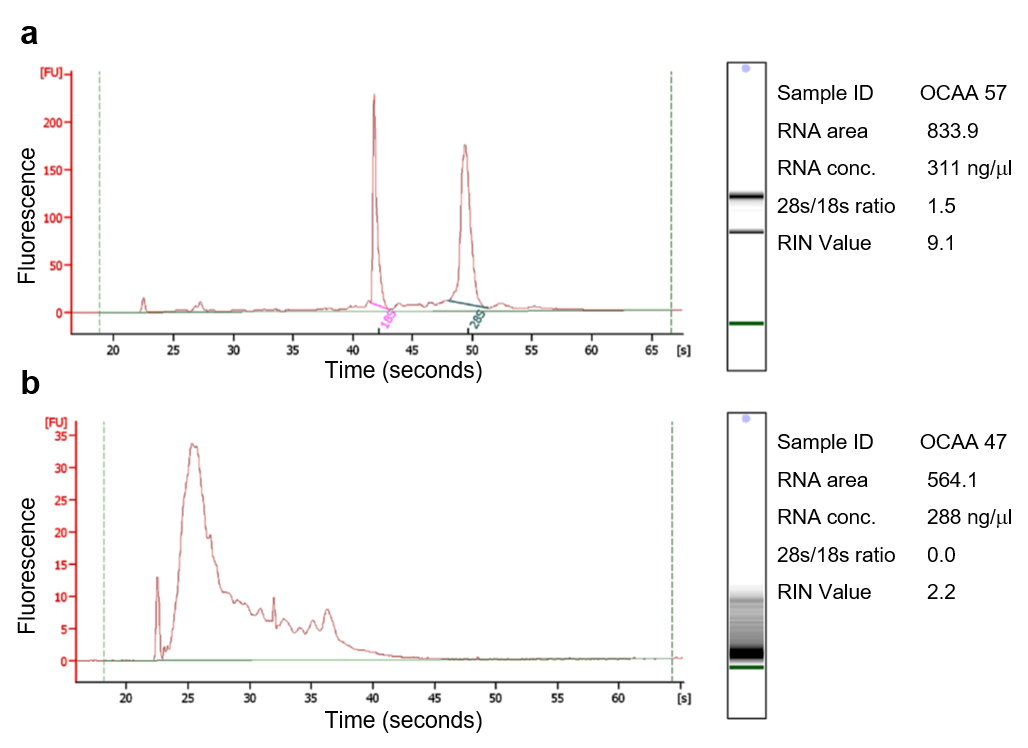
**

(a). Good quality RNA: The fluorescence peaks observed at 0-45 sec and at 50 sec and the RIN value of 9.1 indicate the integrity of the sample. Such samples were included in microarray profiling (b). Degraded RNA: The fluorescence peaks observed at 25-35 sec and the RIN value of 2.2 is indicative of the low molecular weight degraded RNA. Such samples were excluded from microarray profiling.
