# Supplementary material for: Oral squamous cell carcinoma: microRNA expression profiling and integrative analyses for elucidation of tumourigenesis mechanism
Source: Mol Cancer. 2016 Apr 7;15:28. doi: 10.1186/s12943-016-0512-8 (PMC4823852; doi:10.1186/s12943-016-0512-8)
Supplement: Additional file 2: — Details of miRNA assays used in the study. (DOCX 14 kb) [file 12943_2016_512_MOESM2_ESM.docx]

**Additional File 1: Details of miRNA assays used in the study**

| Assay ID* | Assay Name | miRBase ID | Sequence |
| --- | --- | --- | --- |
| miRNA assays | | | |
| 000377 | hsa-let-7a | hsa-let-7a-5p | UGAGGUAGUAGGUUGUAUAGUU |
| 002283 | hsa-let-7d | hsa-let-7d-5p | AGAGGUAGUAGGUUGCAUAGUU |
| 000382 | hsa-let-7f | hsa-let-7f-5p | UGAGGUAGUAGAUUGUAUAGUU |
| 000391 | hsa-miR-16 | hsa-miR-16-5p | UAGCAGCACGUAAAUAUUGGCG |
| 000413 | hsa-miR-29b | hsa-miR-29b-3p | UAGCACCAUUUGAAAUCAGUGUU |
| 000464 | hsa-miR-142-3p | hsa-miR-142-3p | UGUAGUGUUUCCUACUUUAUGGA |
| 002676 | hsa-miR-144 | hsa-miR-144-3p | UACAGUAUAGAUGAUGUACU |
| 000507 | hsa-miR-203 | hsa-miR-203a | GUGAAAUGUUUAGGACCACUAG |
| 002295 | hsa-miR-223 | hsa-miR-223-3p | UGUCAGUUUGUCAAAUACCCCA |
| 002840 | hsa-miR-1275 | hsa-miR-1275 | GUGGGGGAGAGGCUGUC |
| Endogenous reference RNAs | | | |
| 001094 | RNU44 | NR_002750# | CCTGGATGATGATAGCAAATGCTGACTGAACATGAAGGTCTTAATTAGCTCTAACTGACT |
| 001006 | RNU48 | NR_002745# | GATGACCCCAGGTAACTCTGAGTGTGTCGCTGATGCCATCACCGCAGCGCTCTGACC |
| 000407 | hsa-miR-26b | hsa-miR-26b-5p | UUCAAGUAAUUCAGGAUAGGU |
| 001973 | U6 snRNA | NR_004394# | GTGCTCGCTTCGGCAGCACATATACTAAAATTGGAACGATACAGAGAAGATTAGCATGGCCCCTGCGCAAGGATGACACGCAAATTCGTGAAGCGTTCCATATTTT |

*All assay IDs follow the Inventoried catalog no. 4427975. # NCBI Accession number.
