# Supplementary material for: Oral squamous cell carcinoma: microRNA expression profiling and integrative analyses for elucidation of tumourigenesis mechanism
Source: Mol Cancer. 2016 Apr 7;15:28. doi: 10.1186/s12943-016-0512-8 (PMC4823852; doi:10.1186/s12943-016-0512-8)
Supplement: Additional file 4: — Association testing of the 2 major clusters of OSCC samples with their clinico-pathological characteristics. (DOCX 15 kb) [file 12943_2016_512_MOESM4_ESM.docx]

**Additional File 4: Association testing of the 2 major clusters of OSCC samples with their clinico-pathological characteristics**

| **Clinical parameter** | **Group I** | **Group II** | ***P*-value** |
| --- | --- | --- | --- |
| ***Mean age ± SD*** | 56.82±12.54 | 55.17±11.44 | 0.72 |
| ***Gender*** |  |  | 1.00 |
| Male | 05 | 08 |  |
| Female | 06 | 10 |  |
| ***Anatomical site*** |  |  | 1.00 |
| Gingivo-Buccal complex | 08 | 14 |  |
| Tongue | 03 | 04 |  |
| ***Tumor stage*** |  |  | 0.36 |
| T2 | 01 | 04 |  |
| T3 and T4 | 09 | 10 |  |
| Status unknown* | 01 | 04 |  |
| ***Nodal invasion*** |  |  | 1.00 |
| Negative (N-) | -- | 01 |  |
| Positive (N+) | 10 | 13 |  |
| Status unknown* | 01 | 04 |  |
| ***Histological grade*** |  |  | 0.72 |
| G1 (well differentiated) | 04 | 08 |  |
| G2 (moderately differentiated | 07 | 10 |  |
| ***Risky habit profile*** |  |  | 0.15 |
| Any risky habit | 08 | 15 |  |
| No risky habit | 02 | -- |  |
| Status unknown* | 01 | 03 |  |
| ***Smoking*** |  |  | 0.38 |
| Yes | 04 | 03 |  |
| No  Status unknown* | 06  01 | 12  03 |  |
| ***Tobacco chewing*** |  |  | 0.67 |
| Yes | 06 | 11 |  |
| No  Status unknown* | 04  01 | 04  03 |  |
| ***Alcohol consumption*** |  |  | 1.00 |
| Yes | 04 | 05 |  |
| No  Status unknown* | 06  01 | 10  03 |  |

SD – Standard deviation. **P*-value calculated after the missing values (status unknown) were excluded.
