# Supplementary material for: Oral squamous cell carcinoma: microRNA expression profiling and integrative analyses for elucidation of tumourigenesis mechanism
Source: Mol Cancer. 2016 Apr 7;15:28. doi: 10.1186/s12943-016-0512-8 (PMC4823852; doi:10.1186/s12943-016-0512-8)
Supplement: Additional file 5: — Clinico-pathological characteristics of the 4 subgroups of oral cancer samples based on unsupervised clustering of microarray data. (DOCX 14 kb) [file 12943_2016_512_MOESM5_ESM.docx]

**Additional File 5: Clinico-pathological characteristics of the 4 subgroups of oral cancer samples based on unsupervised clustering of microarray data**

| **Clinical parameter** | **Group IA** | **Group IB** | **Group IIA** | **Group IIB** |
| --- | --- | --- | --- | --- |
| ***Mean age ± SD*** | **38.5±9.19** | 60.89±9.13 | 59.22±12.67 | 51.11±8.99 |
| ***Gender*** |  |  |  |  |
| Male | 2 | 3 | 3 | 4 |
| Female | 0 | 6 | 6 | 5 |
| ***Anatomical site*** |  |  |  |  |
| Gingivo-Buccal complex | 0 | 8 | 8 | 6 |
| Tongue | 2 | 1 | 1 | 3 |
| ***Tumor stage*** |  |  |  |  |
| T2 | 0 | 1 | 1 | 3 |
| T3 and T4 | 2 | 7 | 6 | 4 |
| Status unknown |  | 1 | 2 | 2 |
| ***Nodal invasion*** |  |  |  |  |
| Negative (N-) | 0 | 0 | 0 | 1 |
| Positive (N+) | 2 | 8 | 7 | 6 |
| Status unknown |  | 1 | 2 | 2 |
| ***Histological grade*** |  |  |  |  |
| G1 (well differentiated) | 0 | 4 | **7** | **1** |
| G2 (moderately differentiated) | 2 | 5 | **2** | **8** |
| ***Risky habit profile*** |  |  |  |  |
| Any risky habit | 2 | 6 | 8 | 7 |
| No risky habit | 0 | 2 | 0 | 0 |
| Status unknown |  | 1 | 1 | 2 |
| ***Habit of smoking*** |  |  |  |  |
| Yes | 2 | 2 | 2 | 1 |
| No | 0 | 6 | 6 | 6 |
| ***Habit of chewing*** |  |  |  |  |
| Yes | 1 | 5 | 6 | 5 |
| No | 1 | 3 | 2 | 2 |
| ***Alcohol consumption*** |  |  |  |  |
| Yes | 2 | 2 | 2 | 3 |
| No | 0 | 6 | 6 | 4 |

SD – Standard deviation.
