# Supplementary material for: Oral squamous cell carcinoma: microRNA expression profiling and integrative analyses for elucidation of tumourigenesis mechanism
Source: Mol Cancer. 2016 Apr 7;15:28. doi: 10.1186/s12943-016-0512-8 (PMC4823852; doi:10.1186/s12943-016-0512-8)
Supplement: Additional file 6: — Volcano plot of the supervised clustering analysis of microarray data. (DOCX 92 kb) [file 12943_2016_512_MOESM6_ESM.docx]

**Additional File 6: Volcano plot of the supervised clustering analysis of microarray data.**

**
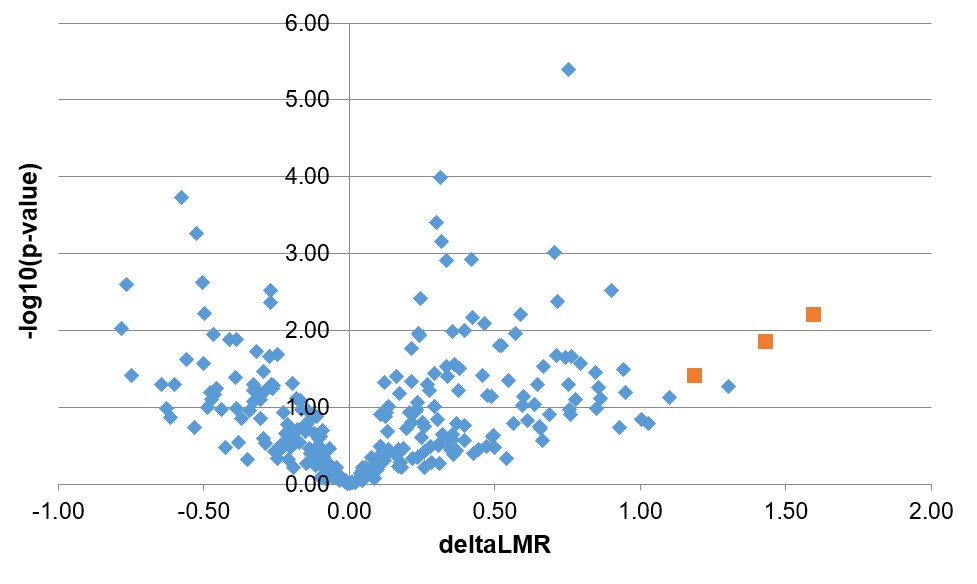
**

This volcano plot shows the relation between the logarithm of the p-values and the deltaLogMedianRatio (dLMR). The *blue* dots represent insignificant miRNAs while *orange* dots represent miRNAs that qualified the fold-change and P-value cut-offs.
