# Supplementary material for: Oral squamous cell carcinoma: microRNA expression profiling and integrative analyses for elucidation of tumourigenesis mechanism
Source: Mol Cancer. 2016 Apr 7;15:28. doi: 10.1186/s12943-016-0512-8 (PMC4823852; doi:10.1186/s12943-016-0512-8)
Supplement: Additional file 7: — Box and whisker plot of the Ct values and their corresponding amplification plot of the 4 reference small RNAs. (DOCX 314 kb) [file 12943_2016_512_MOESM7_ESM.docx]

**Additional File 7: Box and whisker plot of the C_t_ values and their corresponding amplification plot of the 4 reference small RNAs.**


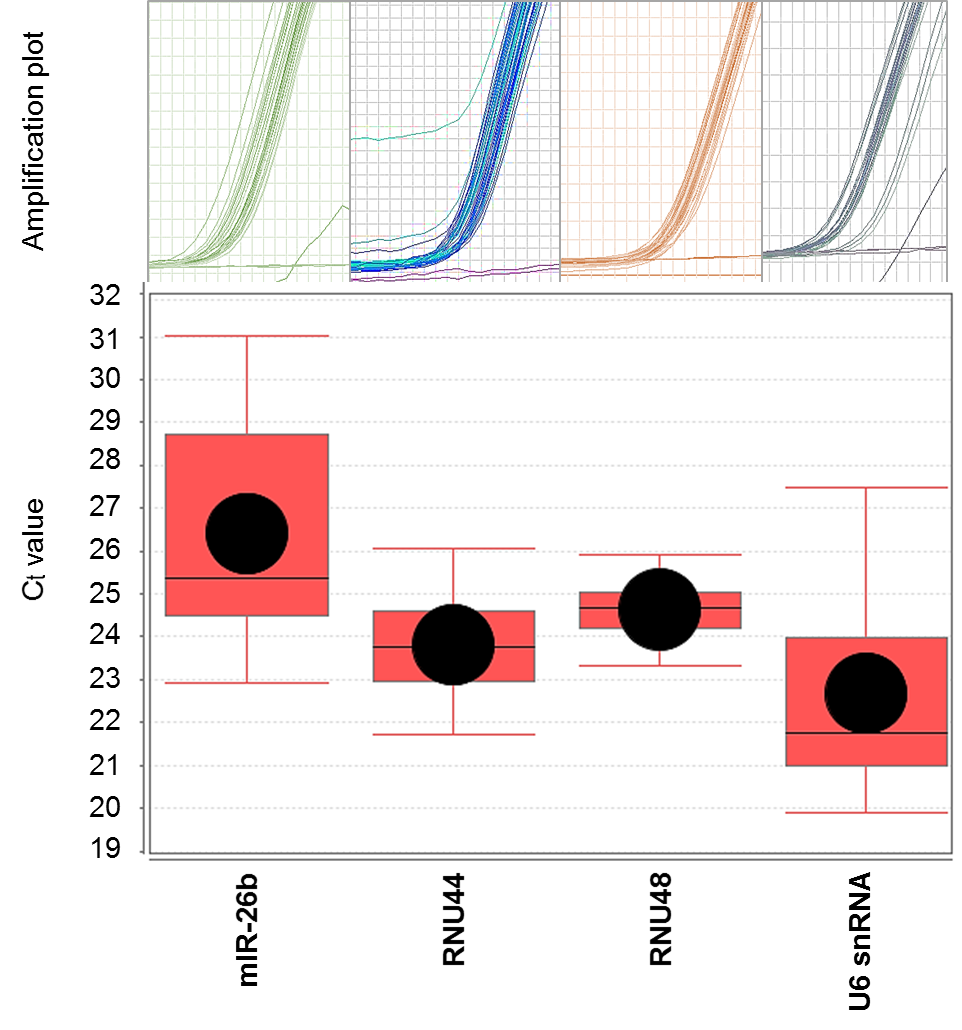


The Box and whisker plot represent the range of the C_t_ values observed for a particular reference RNA across the 10 OSCC and 2 control specimens, with median marked in the center. The corresponding amplification plot of each Reference RNA is shown on the top.
