# Supplementary material for: Oral squamous cell carcinoma: microRNA expression profiling and integrative analyses for elucidation of tumourigenesis mechanism
Source: Mol Cancer. 2016 Apr 7;15:28. doi: 10.1186/s12943-016-0512-8 (PMC4823852; doi:10.1186/s12943-016-0512-8)
Supplement: Additional file 8: — Consolidated clinico-pathological profile of the 61 OSCC samples included in RT-qPCR validation. (DOCX 14 kb) [file 12943_2016_512_MOESM8_ESM.docx]

**Additional File 8: Consolidated clinico-pathological profile of the 61 OSCC samples included in RT-qPCR validation**

| **Clinical parameter** | **No. of patients (Percentage)** |
| --- | --- |
| ***Age*** |  |
| Mean ±SD | 54.69±11.33 |
| ≤ 60 yrs. | 48 (78.7) |
| > 60 yrs. | 13 (21.3) |
| ***Gender*** |  |
| Male | 26 (42.6) |
| Female | 35 (57.4) |
| ***Anatomical site*** |  |
| Alveolar ridge | 8 (13.1) |
| Buccal mucosa | 33 (54.1) |
| Floor of mouth | 5 (08.2) |
| Hard palate | 2 (03.3) |
| Lip | 2 (03.3) |
| Retromolar trigone | 1 (01.6) |
| Tongue | 10 (16.4) |
| ***Clinical stage*** |  |
| I or II | 4 (06.6) |
| III or IV | 47 (77.0) |
| Status unknown | 10 (16.4) |
| ***Regional lymph node metastasis*** |  |
| N0 | 6 (9.8) |
| N1 | 20 (32.8) |
| N2 | 20 (32.8) |
| N3 | 5 (08.2) |
| Status unknown | 10 (16.4) |
| ***Histological grade*** |  |
| G1 (well differentiated) | 23 (37.7) |
| G2 (moderately differentiated) | 34 (55.8) |
| G3 (poorly differentiated) | 1 (01.6) |
| Status unknown | 3 (04.9) |
| ***Risky habit profile*** |  |
| Exclusive smokers | 4 (06.6) |
| Exclusive tobacco chewers | 29 (47.5) |
| Exclusive alcohol drinkers | 1 (01.6) |
| Mixed habitués* | 14 (23.0) |
| None | 6 (09.8) |
| Status unknown | 7 (11.5) |

SD – Standard deviation. *Mixed habitué’s: Patients with at least two of the habits of smoking, chewing or drinking.
