# Supplementary material for: Oral squamous cell carcinoma: microRNA expression profiling and integrative analyses for elucidation of tumourigenesis mechanism
Source: Mol Cancer. 2016 Apr 7;15:28. doi: 10.1186/s12943-016-0512-8 (PMC4823852; doi:10.1186/s12943-016-0512-8)
Supplement: Additional file 9: — CytoScape representation of the miRNA:mRNA interaction network. (DOCX 1528 kb) [file 12943_2016_512_MOESM9_ESM.docx]

**Additional File 9: Cytoscape representation of the miRNA:mRNA interaction network.**

**
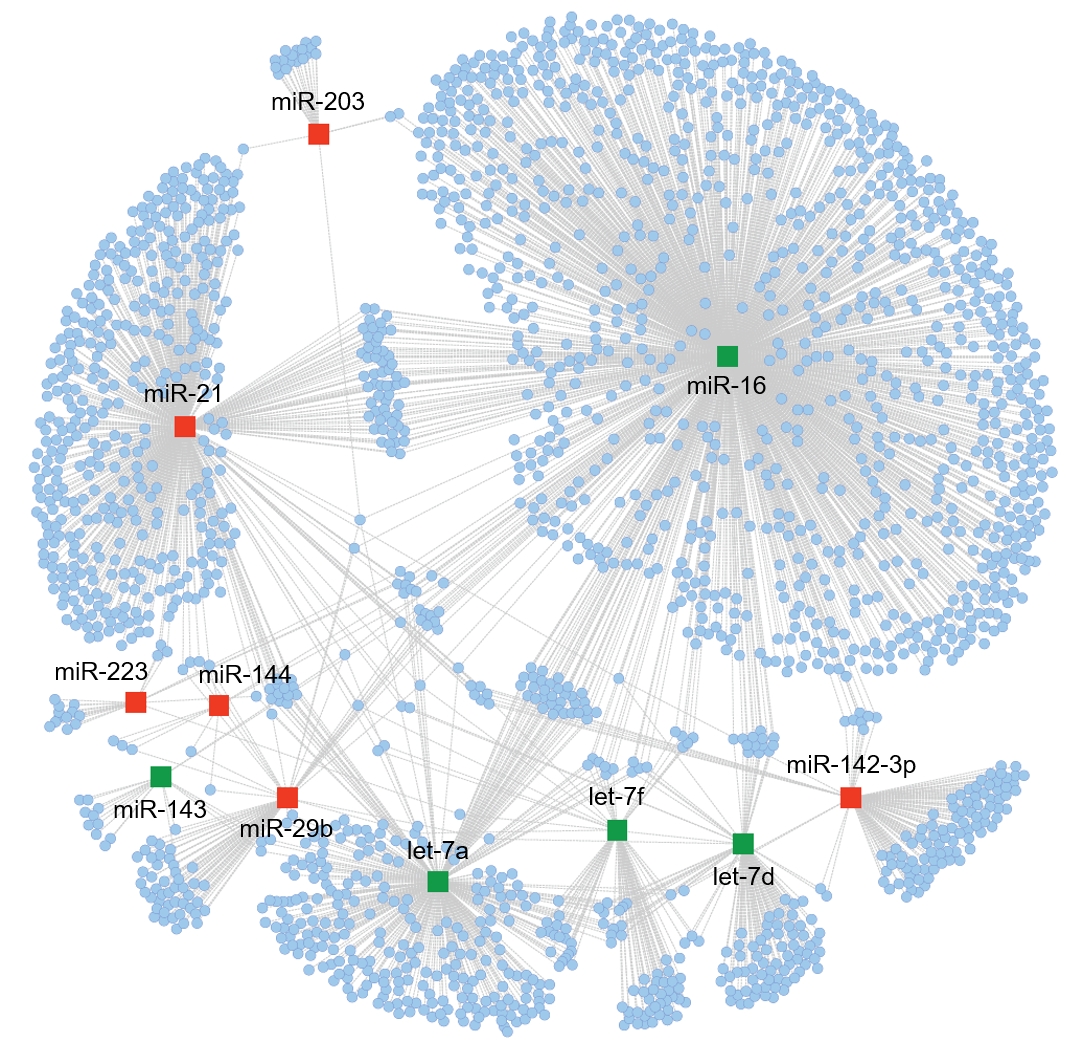
**

*Green* squares (n = 5) represent down regulated miRNAs while *red* squares (n = 6) represent over expressed miRNAs. The *blue* dots (n = 2121) represent the validated targets of these miRNAs. The lines connecting the miRNAs and genes represent the interactions between them.
